# Prevalence and molecular characterization of extended-spectrum β–lactamase—producing *Escherichia coli* isolates from dairy cattle with endometritis in Gansu Province, China

**DOI:** 10.1186/s12917-023-03868-x

**Published:** 2024-01-09

**Authors:** Kang Zhang, Haipeng Feng, Jingyan Zhang, Zhiting Guo, Zunxiang Yan, Guibo Wang, Xuezhi Wang, Lei Wang, Jianxi Li

**Affiliations:** 1 Lanzhou Institute of Husbandry and Pharmaceutical Sciences, Chinese Academy of Agricultural Sciences, Lanzhou, 730050 China; 2 Department of Veterinary Sciences, Gansu Agricultural University, Lanzhou, 730050 China

**Keywords:** *Escherichia coli*, Prevalence, Molecular characterization, Dairy cattle, Endometritis, Extended-spectrum β-lactamase

## Abstract

**Background:**

The present study aimed to investigate the prevalence and molecular characterization of extended-spectrum β-lactamase (ESBL)—producing *Escherichia coli* (*E. coli*) isolated from dairy cattle with endometritis in China. The prevalence of ESBL-producing *E. coli* in sample was detected using ChromID ESBL agar, and genotyping of the ESBL producers was performed by PCR and DNA sequencing.

**Results:**

The results revealed that the proportion of positive pathogens tested was 69.76% (180/258) in samples obtained from cows diagnosed with clinical endometritis, with *E. coli* accounting for 170 out of the 180 positive samples. The infection rate of isolated *E. coli w*as 39.14% (101/258), and co-infections with other pathogens were prevalent. Furthermore, among the 158 *E. coli* isolates, 50 strains were identified as ESBL producers, with TEM and CTX-M prevalence rates at 78.00% and 32.00%, respectively. Drug sensitivity experiments indicated that 50 isolates of ESBL- producing *E. coli* were multidrug resistance (MDR), with 48.0% of them exhibiting positive results for both the class 1 integron gene and five gene cassettes associated with resistance to trimethoprim (dfr1 and dfrA17) and aminoglycosides (aadA1, aadA5, and dfrA1), respectively.

**Conclusion:**

This investigation demonstrated a substantial prevalence and heightened level of antimicrobial resistance among ESBL-producing *E. coli* isolates derived from dairy cattle infected with endometritis in China.

**Supplementary Information:**

The online version contains supplementary material available at 10.1186/s12917-023-03868-x.

## Background

Endometritis is one of the most important reproductive diseases that damages the reproductive performance of cows around the world [1]. Bacterial infection is primary pathogenic factor that caused endometritis in cows, previous studies reported that *Escherichia coli* (*E. coli*), *Streptococcus*, and *Staphylococcus* are the most common pathogens in cow breeding farms [2–4]. The effective treatment measures for endometritis in dairy cows remain the intrauterine infusion of antibiotics. However, there is a gradual increase in the prevalence of multidrug resistance (MDR) strains of *E. coli* due to prolonged and irregular antibiotic usage, accompanied by continuously strengthening level of drug resistance [5, 6]. Therefore, the investigation on the prevalence of *E. coli* strains in dairy farms and their impact on β-lactamase drug resistance is of great significance for maintaining healthy dairy farming practice and reducing economic losses.

Bacteria that carry genes encoding extended-spectrum β-lactamases (ESBLs) have the ability to enzymatically break down a wide range of β-lactamase antibiotics, including penicillins and cephalosporins. *E. coli* strains that produce ESBLs exhibited MDR, which suggested that they are not only resistant to β-lactam antibiotics but also non-β-lactam antibiotics. The extensive MDR poses significant challenges in the clinical management of both human and animal *E. coli* infection [7–9]. Previous studies have indicated that the ESBL genes are responsible for resistance encompass CTX-M, TEM, and SHV, which are easily transmissible and promote the dissemination of resistance genes. In China, the CTX-M-type strain is the most predominant ESBLs, with a predominance of CTX-M-14 [10, 11]. The recent study has revealed that integron, which is hereditary units possessing gene capture and expression function, plays a pivotal role in the mechanism of bacteria resistance [12]. The prevalence of integron, especially class 1 integron, among multidrug resistant *E. coli* strains facilitates the horizontal transfer of resistance genes as mobile genetic elements, which contributes to the widespread emergence of MDR [13]. Extensive researches have been conducted on *E. coli* integrons and gene cassettes. However, the majority of these studies have primarily focused on non-pathogenic strains that isolated from healthy animals or the environment, with limited attention given to investigating pathogenic strains [14–17]. Interestingly, the majority of ESBL-producing *E. coli* strains were predominantly identified within cattle herds comprising less than or equal to 2, 000 cows, potentially indicated a lack of knowledge and skills among producers associated with these farms [18]. Therefore, there are a dearth of comprehensive reports on the molecular characterization of ESBL producing *E. coli* isolates that is associated with endometritis in cattle from large-scale dairy farms located in western of China.

The objective of this study was to detect the prevalence and characterize ESBL- producing *E. coli* strains isolated from bovine endometritis cases in Gansu province recently, then the drug resistance pattern, the associations between resistance phenotypes and genotypes were explored. Our results provided valuable insights for preventing and controlling *E. coli* infection in both livestock, as well as providing novel therapeutic strategies in future research.

## Results

### The identification of the causative agent responsible for endometritis

**Fig. 1:**
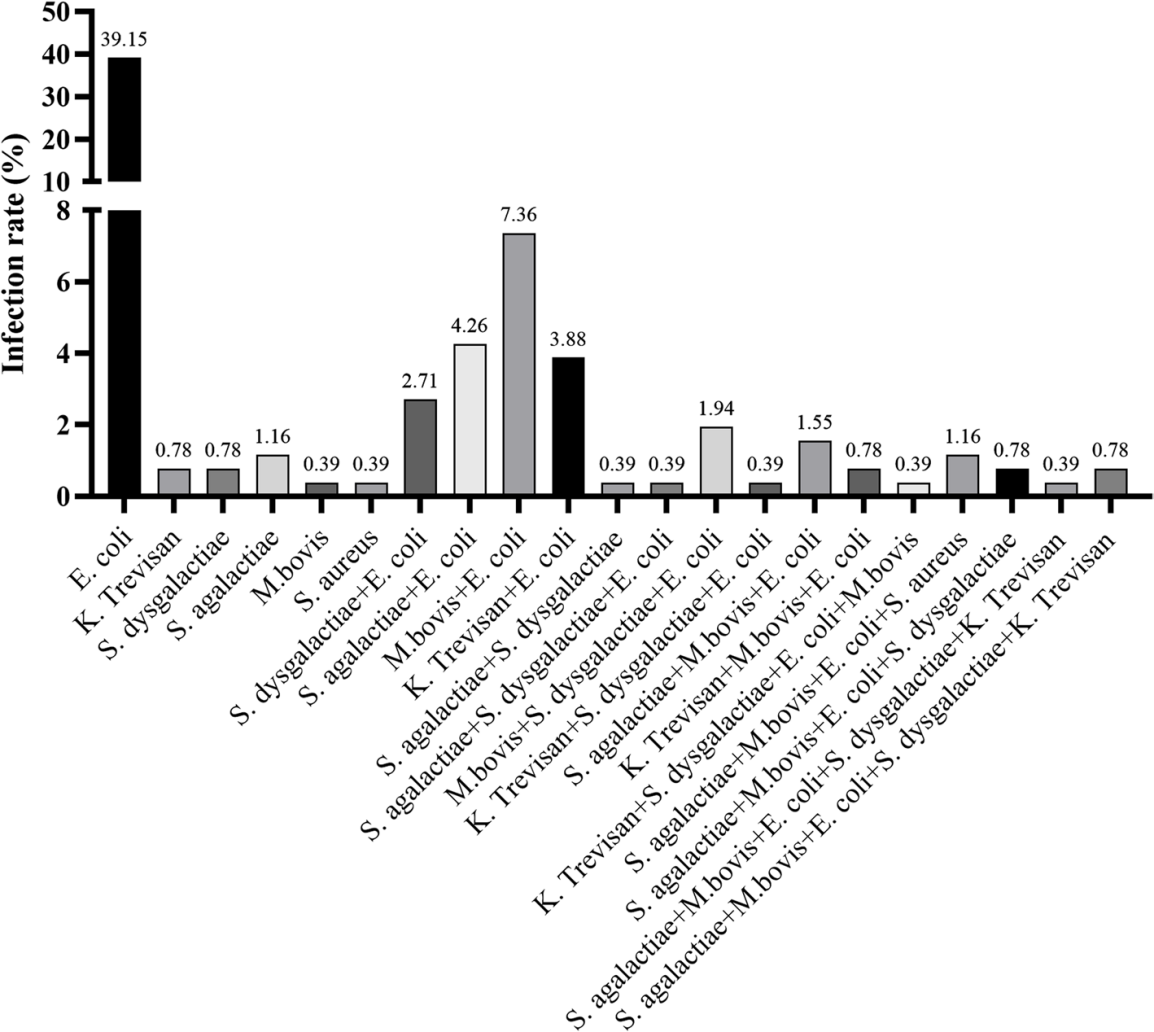
Pathogens causing endometritis were detected in uterine secretions of all clinical cattle using PCR. The ratio of individual or combined pathogens was analyzed by a Chi-square test

The uterine secretions that diagnosed with clinical endometritis were collected from 258 cows, and pathogen detection was performed using PCR. The results revealed that diverse pathogens were detected with the percentage of 69.76% (180/258) in collected samples, with *E. coli* being identified in 170 out of the 180 positive samples. The infection rate of isolated *E. coli* was 39.14% (101/258), while the co-infection rate of *E. coli* with *Streptococcus dysgalactiae (S. dysgalactiae)*, *S. agalactiae* (*S. agalactiae*)*, Mycoplasma bovis* (*M. bovis*), *Klebsiella Trevisan* (*K. Trevisan*)*, S. agalactiae* + *Streptococcus dysgalactiae* (*S. dysgalactiae*)*, M.bovis* + *S. dysgalactiae, K. Trevisan* + *S. dysgalactiae, S. agalactiae* + *M.bovis, K. Trevisan* + *M.bovis, K. Trevisan* + *S. dysgalactiae* + *M.bovis, S. agalactiae* + *M.bovis* + *S. aureus, S. agalactiae* + *M.bovis* + *S. dysgalactiae, S. agalactiae* + *M.bovis* + *S. dysgalactiae* + *K. Trevisan, S. agalactiae* + *M.bovis* + *S. dysgalactiae* + *K. Trevisan* was 2.71% (7/258), 4.26% (11/258), 7.36% (19/258), 3.88% (10/258), 0.39% (1/258), 1.94% (5/258), 0.39% (1/258), 1.55% (4/258), 0.78% (2/258), 0.39% (1/258), 1.16% (3/258), 0.78% (2/258), 0.39% (1/258), and 0.78% (2/258) in turn. These results indicated that *E. coli* is one of the most widespread pathogens in dairy farms and co-infection with other pathogens were also common (26.76%), as shown in Fig. 1.

### Isolation and identification of *E. coli* and ESBLs

In this study, a total of 158 clinical strains were isolated from 258 samples in clinical endometritis uterine secretions that obtained from dairy cows. A total of 158 clinical strains were identified as *E. coli* based on colony morphology analysis on blood agar, gram staining, biochemical identification, and 16S rDNA sequencing. Additionally, the ChromID ESBL agar results showed that 23.04% samples were tested positive for ESBL production based on the ChromID ESBL agar culture among these isolates, as shown in Table 1.

**Table 1:** Prevalence of ESBL-producing *Escherichia coli* isolated from Gansu province in China

Herd No	Size	No. of dairy cows with clinical Endometritis	No. of *E. coli*	No. of ESBL producers	ESBL detection rate (%)
1	1200	21	19	6	31.57
2	4557	18	14	3	21.42
3	1845	13	12	5	41.66
4	1011	15	13	4	30.76
5	2147	11	7	2	28.57
6	1754	13	4	2	50.00
7	1552	21	11	5	45.45
8	1962	14	12	4	33.33
9	3468	12	8	0	0.00
10	2885	18	6	2	33.33
11	2011	18	10	2	20.00
12	2235	13	4	2	50.00
13	1758	15	10	3	30.00
14	2250	19	4	0	0.00
15	925	12	9	5	55.55
16	856	14	11	4	36.36
17	1740	11	4	1	25.00

The susceptibility profiles of β-lactamase and the associated antimicrobial resistance of ESBL-producing isolates are summarized in Table 2. All isolates exhibited MDR and were resistant to ampicillin. The majority of isolated strains showed resistance to gentamicin (78%), amoxicillin (66%), ceftiofur (62%), and tetracycline (58%). Notably, a total of 26 isolates (52%) were resistant to all tested penicillins, cephalosporins, and monobactams. Except for β-lactamase resistance testing, these isolates were subjected to antimicrobial susceptibility testing in various categories. It is worth noting that a high level of resistance was observed against tetracycline (58%) as well as erythromycin (56%), trimethoprim (52%), oxytetracycline (50%), enrofloxacin (42%) and streptomycin (38%). Importantly, *E. coli* is intrinsically resistant to erythromycin.

**Table 2:** Antimicrobial resistance patterns of ESBL-producing *E. coli* isolates (*n* = 50) from dairy cattle with endometritis

Antibiotics	Susceptible (number/%)	Intermediate (number/%)	Resistance (number/%)
mpicillin	0/0	0/0	50/100
Amoxicillin	5/10	12/24	33/66
Ceftiofur	3/6	16/32	31/62
Cefotaxime	8/16	15/30	27/54
Trimethoprim	24/48	0/0	26/52
Tetracycline	20/40	1/2	29/58
Gentamicin	8/16	3/6	39/78
Oxytetracycline	24/48	1/2	25/50
Florfenicol	49/98	1/2	0/0
Erythromycin	8/16	14/28	28/56
Meropenem	50/100	0/0	0/0
Imipenem	50/100	0/0	0/0
Streptomycin	31/62	0/0	19/38
Enrofloxacin	19/58	0/0	21/42

### Characterization of ESBLs genes and class 1 integrons

**Fig. 2:**
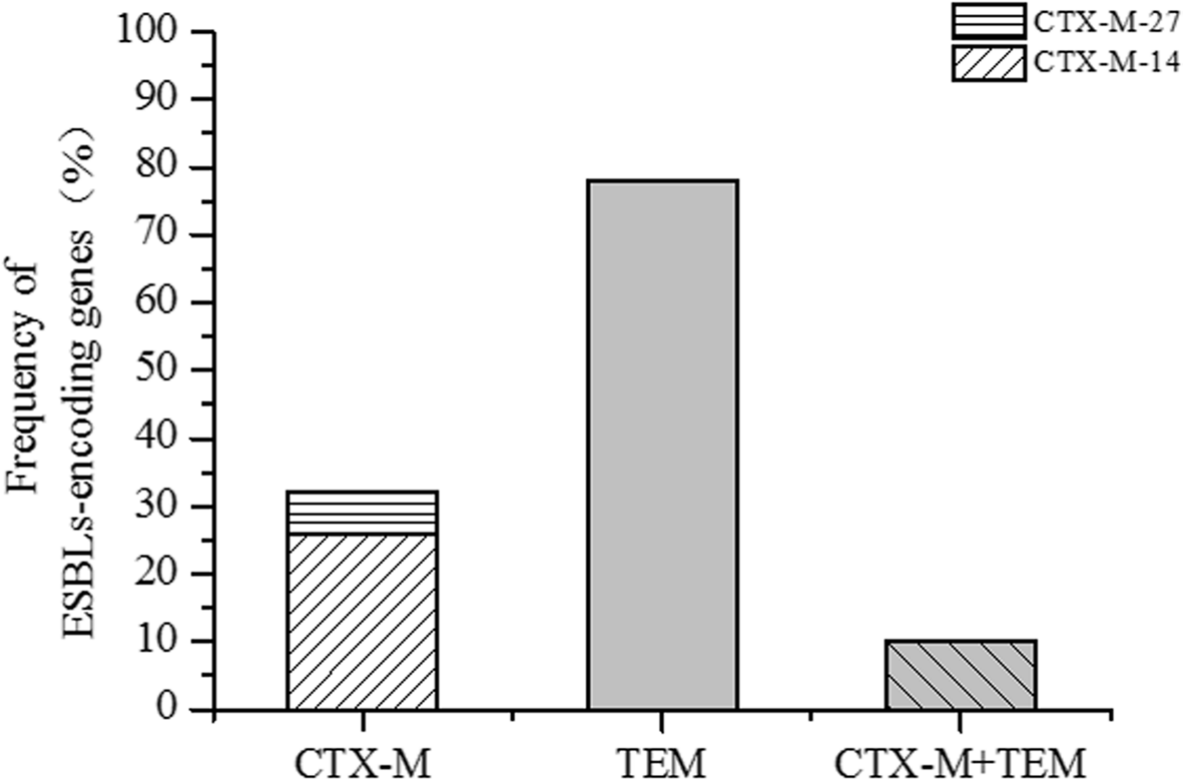
Distribution and subtyping of CTX-M ESBL-encoding genes in *E*. *coli* isolates with ESBL Production

The TEM gene was detected in 78% of the isolates, whereas CTX-M was identified in 32% of the ESBL-producing *E. coli* isolates (16 out of 50). Specifically, CTX-M-14 tested positive in 26% (13 out of 50), and CTX-M-27 exhibited positivity in 6% (3 out of 50), as depicted in Fig. 2. Furthermore, the co-occurrence of TEM and CTX-M genes was observed with 10% (5/50) in the *E. coli* isolates, while SHV gene was not detected in all isolates. Moreover, the presence of intI-1 genes was detected in 48% (24/50) of all isolates, while none of the isolates exhibited the occurrence of either intI-2 or intI-3. However, amplification of gene cassettes from three intI1- positive isolates was not successful, indicating the absence of amplifiable genetic material in these isolates. The class 1 integrons contained five distinct gene cassettes: dfrA1, dfrA17-aadA5, aadA1, aadA5 and dfr1-aadA1. Among the gene cassettes identified within these integrons, dfrA17-aadA5 exhibited the highest prevalence (26%, 13 isolates), followed by dfrA1 (10%, 5 isolates), aadA1 (6%, 3 isolates), aadA5 (4%, 2 isolates) and dfr1-aadA1 (4%, 2 isolates), as presented in Table 3.

**Table 3:** Phenotypic antibiotic resistance and the occurrence of integrons in E. *coli* isolates

No. of strain	Resistance phenotypes^a^	Integrons^b^	Gene cassettes^c^
1	AMP/TRI/TET/GEN/OXY/ERY	Class1	dfrA1
2	AMP/AMO/CES/TET	Class1	dfrA1
3	AMP/AMO/CES/TRI/GEN/TET/GEN/OXY/ERY/ENR	Class1	aadA5
4	AMP/AMO/CES/CEF/TRI/GEN	Class1	dfrA17-aadA5
5	AMP/AMO/CES/TRI/GEN	Class1	dfrA17-aadA5
6	AMP/AMO/TET/GEN/OXY	Class1	dfr1-aadA1
7	AMP/AMO/GEN//ERY	Class1	dfrA17-aadA5
8	AMP/AMO/CES/TRI/TET/GEN/OXY/ERY	Class1	dfrA17-aadA5, dfrA1
9	AMP/AMO/CES/CEF/TRI/OXY/ERY	Class1	dfrA1
10	AMP/AMO/CES/CEF/TRI/TET/OXY/ERY/STR	Class1	dfrA17-aadA5
11	AMP/CES/CEF/TRI/GEN/OXY/ERY/STR/ENR	Class1	**undetected**
12	AMP/AMO/CES/CEF/TET/GEN/OXY/STR	Class1	dfrA17-aadA5, aadA1
13	AMP/AMO/CES/CEF/TRI/TET/GEN/STR	Class1	dfrA17- aadA5
14	AMP/AMO/CES/CEF/TRI/GEN/ERY	Class1	aadA1
15	AMP/AMO/CES/CEF/TRI/GEN/OXY	Class1	dfrA17-aadA5
16	AMP/AMO/TRI/TET/GEN/STR/ENR	Class1	dfrA1
17	AMP/TRI/TET/GEN/OXY/ERY/ENR	Class1	**undetected**
18	AMP/AMO/CES/CEF/GEN/OXY/STR	Class1	dfr1-aadA1
19	AMP/AMO/CES/CEF/TET/GEN/OXY/ERY	Class1	dfrA17-aadA5, aadA5
20	AMP/AMO/CES/CEF/TRI/TET/GEN/OXY/ERY	Class1	dfrA17-aadA5
21	AMP/AMO/CES/CEF/TRI/GEN/OXY/ERY/STR	Class1	aadA1, dfrA17-aadA5
22	AMP/AMO/CES/CEF/TRI/TET/GEN/OXY	Class1	**undetected**
23	AMP/AMO/CES/CEF/TET/GEN/OXY/STR/ENR	Class1	dfrA17-aadA5
24	AMP/AMO/CES/CEF/GEN/OXY/ERY/STR/ENR	Class1	dfrA17-aadA5

*AMP* Ampicillin, *AMO* Amoxicillin, *CES* Ceftiofur, *CEF* Cefotaxime, *TRI* Trimethoprim, *TET* Tetracycline, *GEN* Gentamicin, *OXY* Oxytetracycline, *FLO* Florfenicol, *ERY* Erythrouycin, *MER* Meropenem, *IMI* Imipenem, *STR* Streptomycin, *ENR* Enrofloxacin, *aadA1* aminoglycoside-3’-adenylytransferase gene, *dfrA1* dihydrofolate reductase gene, *dfrA17-aadA5* dihydrofolate reductase gene and aminoglycoside-3’-adenylytransferase gene, *dfr1-aadA1* dihydrofolate reductase gene and aminoglycoside-3’-adenylytransferase gene

^a^The most common multi-drug resistance pattern observed among *E*. coli isolates

^b^Integrons, Class1 integron gene

^c^Kinds of gene cassettes

## Discussion

Endometritis is a prevalent perinatal ailment in dairy cattle, which serves as the primary cause of bovine infertility and significantly hinders the progress of the dairy cattle breeding industry. Pathogenic bacterial infection emerges as a prominent factor contributing to cow endometritis, with mixed infections involving multiple pathogenic bacteria being the primary etiological determinant of this disease [19–21]. The present study systematically investigated pathogenic microorganisms associated with cow endometritis in Gansu Province, China. The results indicated that bovine Endometritis caused by *E. coli* exhibited multiple drug resistance, and E. *coli* and ESBLs in cows are the dominant strains in western China. These findings provided valuable insights for the prevention and control of *E. coli* infection in livestock and poultry, as well as for the exploration of innovative treatment strategies.

Currently, conventional pathogen detection methods such as culture medium plate coating and ordinary PCR are plagued by issues of time-consuming procedures, instrument limitations, and the need for specialized personnel. Therefore, high-throughput rapid pathogen detection is crucial for early prevention, control, and treatment in cow endometritis. In this study, a total of 258 endometritis cow samples were detected using a nucleic acid test kit for multiple pathogens, and the predominant pathogens were identified. The findings revealed that *E. coli* emerged as the predominant pathogen, constituting the largest proportion among all pathogenic microorganisms. Both single and mixed infections were observed, with a lower incidence of mixed infections compared to those caused by *E. coli* alone. Furthermore, *Mycoplasma* bovis exhibits a significantly elevated detection rate, thereby presenting substantial challenges in the management of mixed infections in cattle and requiring considerable attention from livestock farms.

The β-lactam antibiotics have been used to treat various infectious diseases in humans and animals since the 1940s. However, the emergence of bacterial resistance has led to a decreased trend in the clinical efficacy of these antibiotics, particularly against *Enterobacteriaceae*. As a result, there has been an increase in the number of ESBL-producing *E. coli* strain [22, 23]. In this study, a total of 217 *E. coli* isolates were collected from the case of bovine endometritis in China, and the prevalence of ESBL producers observed in this study was found to be 23.04%, which is consistent with previous studies conducted on bovine *E. coli* in China, while surpassing the reported values from other countries [24–27]. Previous researches confirmed that CTX-M- type ESBLs have replaced TEM and SHV-type ESBLs as the predominant type among *Enterobacteriaceae* members in Asia, Europe, and Canada [28–30]. Our findings revealed that the TEM genotype was the most prevalent, followed by CTX-M in the western of China. Our study has identified the presence of CTX-M-14 and CTX-M-27 producers among bovine- associated *E*. *coli* isolates, which is a novel finding, these producers have previously been reported to be frequently found in clinical isolates of *E. coli* from healthcare facilities [31]. Furthermore, the use of *Meropenem* and *imipenem* in animals has been prohibited, and all ESBL-producing *E*. *coli* isolates have shown susceptibility to these two antibiotics. Our findings are line with previous study that indicated the majority of ESBL producers identified from livestock exhibit MDR [32]. Specifically, we also observed that all ESBL-producing *E*. *coli* isolates were MDR and most were resistant to aminoglycosides, trimethoprim, tetracyclines, chloramphenicol or quinolones.

The prevalence rate of positive integrons in this study was determined to be 48%, which aligns with the reported value (45.5%) observed in Inner Mongolia, China. The variable regions within class 1 integron gene cassette arrays exhibited five distinct gene combinations, which are likely responsible for conferring additional resistance traits onto our isolates. Notably, three intI1-positive amplicons were unable to produce any gene cassettes potentially due to the lack of a 3’-CS region within these particular integrons [33]. The cassette dfrA17-aadA5 was identified as the predominant gene array, which is consistent with previous study conducted in China [34]. The presence of class 1 integrons in pathogenic bacteria has been found to be closely associated with bacterial resistance according to recent research [35, 36]. The variable region of Class 1 integrons exhibited a remarkable level of gene diversity and demonstrated a strong association with resistant phenotypes. Further comprehensive investigations about class 1 integrons can provide valuable insights into the occurrence and dissemination of pathogenic bacteria, thereby establishing a foundation for effective prevention and control measures against epidemics, bacterial infections in breeding enterprises, as well as facilitating the selection of antimicrobial agents.

## Conclusion

In summary, the investigation has revealed that ESBL-producing *E*. *coli* isolates obtained from dairy cattle with endometritis in China exhibited a significant prevalence and elevated antimicrobial resistance level, thereby establishing a theoretical foundation for the prevention and treatment of bovine endometritis.

## Materials and methods

### Sample collection area and experimental animals

**Fig. 3:**
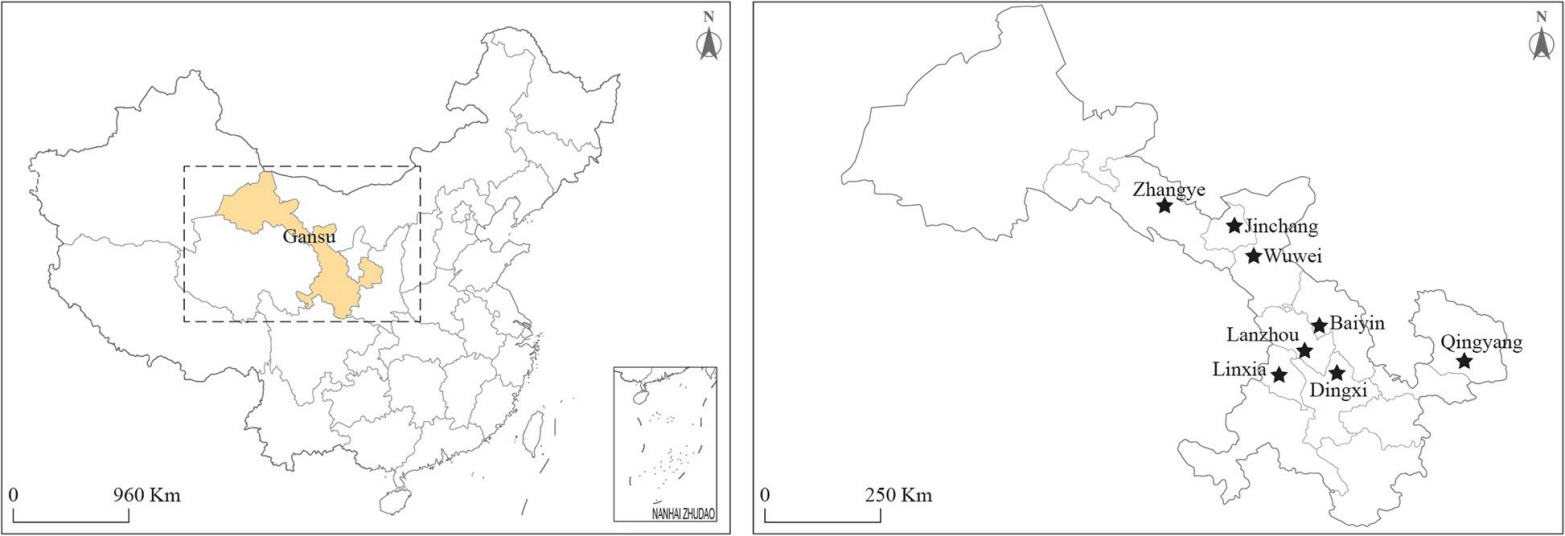
Location of Gansu in China and all the dairy herds surveyed in cities in Gansu province (★Sampling city)

The dairy cows selected in this study were obtained from a total of 17 dairy herds located in Gansu Province, as shown in Fig. 3. These cows ranged in age from 3 to 6 years old, with weights ranging between 650 and 700 kg. None of the participants had a history of retained placenta or septicemia, although some may have encountered initial postpartum challenges such as delayed uterine involution and endometritis. The cows in these 17 dairy farms were vaccinated against BVDV and foot-and-mouth disease vaccines according to standard immunization protocol prior to being utilized for uterine fluid collection. The cow utilized in our study originated from different ranch, where it was internally raised and selectively bred by the ranch itself.

### Samples collection and cases definition

In our study, a total of 258 samples of uterine secretions were collected from dairy cows diagnosed with clinical endometritis from Sep 2020 to Feb 2023. The procedure for sample collection is outlined as follows: The perineal areas of the restrained cows were disinfected with a 75% ethyl alcohol solution. Subsequently, a plastic infusion pipette with a protective sheath was inserted into the cranial vagina and carefully guided through the cervix and into the uterus after rupturing the sheath. A sterile saline solution was gently injected and agitated within the uterus before being aspirated for sampling purposes. The volume of collected samples ranged from 5 to 15 mL and was transported on ice for analysis in a laboratory within 24 h (h).

The inflammation of the endometrium, referred to as endometritis, is characterized by the presence of purulent cervical discharge that can be detected in the vagina 21 days or more postpartum. In most cases, no obvious clinical signs were observed and milk production remains normal. Lochia (the presence of malodorous cervical discharge) and rectal palpation (an enlarged, atonic uterus) were evaluated according to the diagnostic method of clinical endometritis, which was made by a veterinarian with extensive experience in treating cow diseases to ensure an accurate clinical diagnosis.

### DNA amplification and sequencing

The Multiplex TaqMan real-time fluorescent quantitative PCR kit (Shenzhen, China) was employed for pathogen detection according to the manufacturer’s instruction, including *E. coli*, *Staphylococcus aureus* (*S. aureus*), *S. agalactiae*, *M. bovis*, *K. Trevisan, S. dysgalactiae* and *Pseudomonas aeruginosa* (*P. aeruginosa*) in clinical endometritis samples. Bovine respiratory tract pathogen nucleic acid detection kit (fluorescence PCR method) was purchased from Shenzhen Anieasy Biotechnology Co., Ltd. In brief, the Bacterial DNA was extracted using the Bacterial DNA Kit (Omega Bio-Tek, USA) following the manufacturer’s instructions. The different solutions were melt on ice after being removed from the reagent kit, add the mixture of samples and PCR reaction fluids. PCR reaction program as follows: Decontamination for 3 min (min) at 50 °C, pre-denaturation for 3 min at 95 °C, amplification for 10 s at 95 °C and signal collection for 30 s at 60 °C (40 cycles). The judgment of the result is shown in Supplemental Table 1.

The TEM, SHV, and CTX genes, which are prominent members of the β-lactamase gene family, were amplified using multiplex PCR with previously described primers and amplification conditions [25]. The integrase gene fragments were specifically amplified using PCR to identify the presence of integrons in all isolates. Subsequently, the detection of class 1, class 2, and class 3 integrons, as well as their associated gene cassettes, was performed following previously reported PCR conditions. PCR products were confirmed by bi-directional sequencing after being purifying with a QIAquick PCR Purification Kit (Qiagen, Hilden, Germany). The DNA sequences obtained were compared with those in GenBank using the BLAST program (https://blast.ncbi.nlm.nih.gov/Blast.cgi). The primers used in this work are listed in Supplemental Table 2.

### The isolation and identification of ESBL-producing *E. coli*

*E. coli* isolated from endometritis cases were identified as strains of ESBL-producing *E. coli* through morphological characterization and biochemical in our laboratory. In brief, the collected samples were inoculated into fresh blood agar plates and incubated for 24 h at 37 °C under aerobic and anaerobic conditions. The colonies were observed after being cultured for 24 h and conducted Gram staining. Then, the typical colonies were purified for secondary cultivation. Purified colonies were inoculated into 2 mL of nutrient broth and cultured at at 37 °C overnight. Then bacterial DNA was extracted with OMEGA genomic DNA extraction kit according to the instructions. 16S rDNA fragments were amplified using 16S rDNA bacterial identification PCR kit, and the nucleic acid electrophoresis was performed. The target fragment was sequenced by Beijing Liuhe Huada Gene Technology Co., Ltd.

### Testing for antimicrobial susceptibility

ESBL-producing *E*. *coli* isolates were tested for susceptibility against 14 antimicrobial agents using the disc diffusion method, following CLSI recommendations [37]. The antibiotics (Oxoid, United Kingdom) that were tested included ampicillin (10 μg), amoxicillin (30 μg), ceftiofur (30 μg), cefuroxime (30 μg), trimethoprim (25 μg), tetracycline (10 μg), gentamicin (10 μg), oxytetracycline (30 μg), florfenicol (30 μg), erythromycin (10 μg), meropenem (10 μg), imipenem (10 μg), streptomycin (10 μg) and enrofloxacin (5 μg). The quality control strain used in this study was *E. coli* ATCC 25922 and exhibiting resistance to three or more antimicrobial categories were categorized as MDR [38].

### Statistical analysis

All experiments were performed with at least three replications. Statistical analysis was performed using Prism 7.0 Software (GraphPad, La, Jolla, CA), and differences are evaluated by Student’s t-test. For all comparisons, *P* < 0.05 was considered statistically significant, *P* < 0.01 was considered to be extremely significant.

### Supplementary Information

**Additional file 1.**

## Data Availability

The data supporting this study’s findings are available on request from the corresponding author.
