# Supplementary material for: Prevalence and molecular characterization of extended-spectrum β–lactamase—producing Escherichia coli isolates from dairy cattle with endometritis in Gansu Province, China
Source: BMC Vet Res. 2024 Jan 9;20:19. doi: 10.1186/s12917-023-03868-x (PMC10777567; doi:10.1186/s12917-023-03868-x)
Supplement: Supplementary file 1 — Additional file 1. [file 12917_2023_3868_MOESM1_ESM.docx]

**
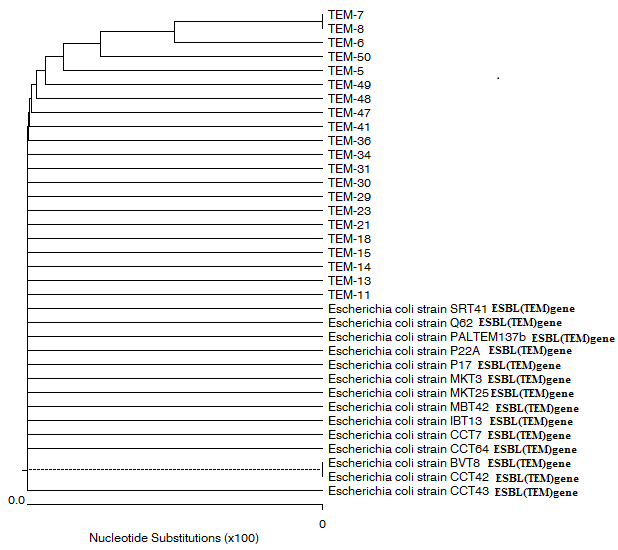
**

**Supplemental Figure1 The evolutionary tree between ESBL TEM standard strain and clinical isolated strain TEM**

**
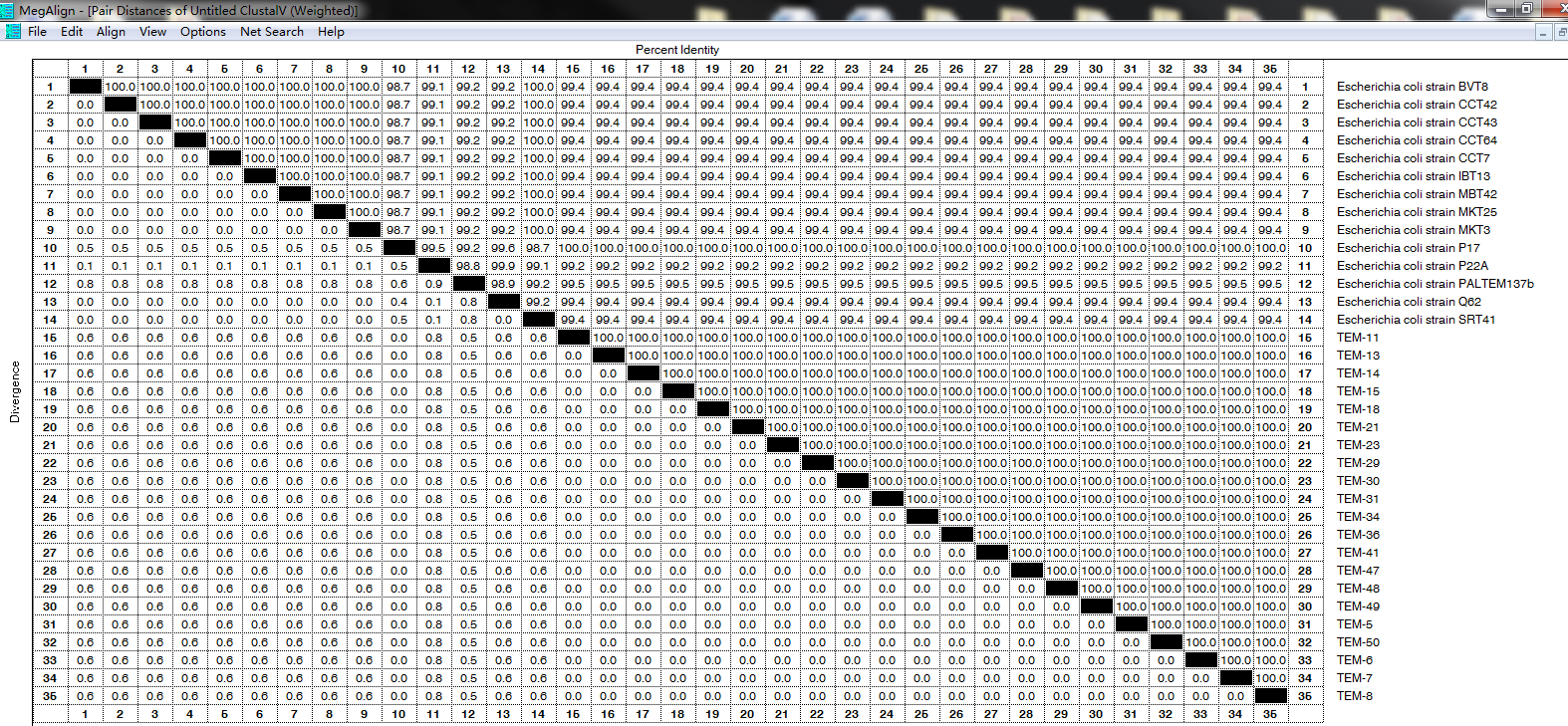
**

**Supplemental Figure2 The homology analysis of TEM-type genes and ESBLs**

**Supplemental Table1 The judgment of the detection kit result**

| Tube number | Fluorescence Channel | item | Strong Positive (+++) | Positive (++) | Weakly positive (+) | Suspected positive (±) | Negative (-) |
| --- | --- | --- | --- | --- | --- | --- | --- |
| 1 | FAM | β Lactamase resistance gene Lac | ≤20 | （20, 28] | (28, 35] | (35, 36] | (36, 40] |
|  | HEX | *Streptococcus agalactis* Sag | ≤25 | （25, 33] | (33, 37] | (37,3 8.5] | (38.5, 40] |
|  | ROX | *Pseudomonas aeruginosa* Pa | ≤22 | （22, 30] | (30, 35] | (35, 38] | (38, 40] |
| 2 | FAM | *Klebsiella* Klsp | ≤20 | （20, 28] | (28, 36] | (36, 38] | (38, 40] |
|  | HEX | *Streptococcus lactis* Sdy | ≤25 | （25, 33] | (33, 37] | (37, 38.5] | (38.5, 40] |
| 3 | FAM | *Staphylococcus aureus* Sau | ≤25 | （25, 33] | (33, 37] | (37, 38.5] | (38.5, 40] |
|  | HEX | *Escherichia coli* Ec | ≤20 | （20, 28] | 28, 35] | (35, 37] | (37, 40] |
|  | ROX | *Mycoplasma bovis* Myb | ≤25 | （25, 33] | (33, 37] | (37, 38.5] | (38.5, 40] |

If there is an amplification signal in the negative control, Ct (negative control) – Ct (Sample) ≥ 3 can be determined as weakly positive.

**Supplemental Table2 The primer sequences for PCR**

| **Gene** | **Primer sequence**  **（5’-3’）** | | **Product size (bp)** | | **Reference** | |
| --- | --- | --- | --- | --- | --- | --- |
| TEM | F: TTTCGTGTCGCCCTTATTCC  R: CCGGCTCCAGATTTATCAGC | | | 692 | Bailey et al., 2011 | |
| SHV | F: GGGTTATTCTTATTTGTCGC  R: TTAGCGTTGCCAGTGCTC | 567 | | | | Chang et al., 2001 |
| CTX-M | F: ATGTGCAGYACCAGTAA  R: ACCGCRATATCRTTGGT | 536 | | | | Amel et al., 2016 |
| intI-1 | F: CCTCCCGCACGATGATC  R: TCCACGCATCGTCAGGC | 280 | | | | Goldstein et al., 2001 |
| intI-2 | F: AAATCTTTAACCCGCAAACGC  R:ATGTCTAACAGTCCATTTTTAAATTCTA | | 439 | | | Dillon et al., 2005 |
| intI-3 | F: AGTGGGTGGCGAATGAGTG  R: TGTTCTTGTATCGGCAGGTG | 599 | | | | Dillon et al., 2005 |
| Int1-VR | F: GGCATCCAAGCAGCAAG  R: AAGCAGACTTGACCTGA | varible region | | | | White et al., 2000 |

Abbreviation: **PCR**, polymerase chain reaction; F, Forward primer; R, Reverse primer.

**Supplemental Table3 The information of gene sequencing data**

| **Name** | **gene sequencing** |
| --- | --- |
| CTX-1 | TATGTGCAGGTCCAGTAAAGTTATGGCGGCCGCGGCGGTGCTTAAGCAGAGTGAAACGCAAAAGCAGCTGCTTAATCAGCCTGTCGAGATCAAGCCTGCCGATCTGGTTAACTACAATCCGATTGCCGAAAAACACGTCAACGGCACAATGACGCTGGCAGAACTGAGCGCGGCCGCGTTGCAGTACAGCGACAATACCGCCATGAACAAATTGATTGCCCAGCTCGGTGGCCCGGGAGGCGTGACGGCTTTTGCCCGCGCGATCGGCGATGAGACGTTTCGTCTGGATCGCACTGAACCTACGCTGAATACCGCCATTCCCGGCGACCCGAGAGACACCACCACGCCGCGGGCGATGGCGCAGACGTTGCGTCAGCTTACGCTGGGTCATGCGCTGGGCGAAACCCAGCGGGCGCAGTTGGTGACGTGGCTCAAAGGCAATACGACCGGCGCAGCCAGCATTCGGGCCGGCTTACCGACGTCGTGGACTGTGGGTGATAAGACCGGCAGCGGCGACTACGGCACCACCAATGATATCGCGGTAAAGG |
| CTX-4 | TATGTGCAAGGTCCAGTAAAGTTATGGCGGCCGCGGCGGTGCTTAAGCAGAGTGAAACGCAAAAGCAGCTGCTTAATCAGCCTGTCGAGATCAAGCCTGCCGATCTGGTTAACTACAATCCGATTGCCGAAAAACACGTCAACGGCACAATGACGCTGGCAGAACTGAGCGCGGCCGCGTTGCAGTACAGCGACAATACCGCCATGAACAAATTGATTGCCCAGCTCGGTGGCCCGGGAGGCGTGACGGCTTTTGCCCGCGCGATCGGCGATGAGACGTTTCGTCTGGATCGCACTGAACCTACGCTGAATACCGCCATTCCCGGCGACCCGAGAGACACCACCACGCCGCGGGCGATGGCGCAGACGTTGCGTCAGCTTACGCTGGGTCATGCGCTGGGCGAAACCCAGCGGGCGCAGTTGGTGACGTGGCTCAAAGGCAATACGACCGGCGCAGCCAGCATTCGGGCCGGCTTACCGACGTCGTGGACTGTGGGTGATAAGACCGGCAGCGGCGACTACGGCACCACCAATGTTAATTCGCGGT |
| CTX-6 | TTATGTGCAGTACCAGTAAAGTTATGGCGGCCGCGGCGGTGCTTAAGCAGAGTGAAACGCAAAAGCAGCTGCTTAATCAGCCTGTCGAGATCAAGCCTGCCGATCTGGTTAACTACAATCCGATTGCCGAAAAACACGTCAACGGCACAATGACGCTGGCAGAACTGAGCGCGGCCGCGTTGCAGTACAGCGACAATACCGCCATGAACAAATTGATTGCCCAGCTCGGTGGCCCGGGAGGCGTGACGGCTTTTGCCCGCGCGATCGGCGATGAGACGTTTCGTCTGGATCGCACTGAACCTACGCTGAATACCGCCATTCCCGGCGACCCGAGAGACACCACCACGCCGCGGGCGATGGCGCAGACGTTGCGTCAGCTTACGCTGGGTCATGCGCTGGGCGAAACCCAGCGGGCGCAGTTGGTGACGTGGCTCAAAGGCAATACGACCGGCGCAGCCAGCATTCGGGCCGGCTTACCGACGTCGTGGACTGTGGGTGATAAGACCGGCAGCGGCGGCTACGGCACCACCAATGATAATCGCGGTAACA |
| CTX-9 | TTTATGTGCAAGGTTCAGTAAAGTTATGGCGGCCGCGGCGGTGCTTAAGCAGAGTGAAACGCAAAAGCAGCTGCTTAATCAGCCTGTCGAGATCAAGCCTGCCGATCTGGTTAACTACAATCCGATTGCCGAAAAACACGTCAACGGCACAATGACGCTGGCAGAACTGAGCGCGGCCGCGTTGCAGTACAGCGACAATACCGCCATGAACAAATTGATTGCCCAGCTCGGTGGCCCGGGAGGCGTGACGGCTTTTGCCCGCGCGATCGGCGATGAGACGTTTCGTCTGGATCGCACTGAACCTACGCTGAATACCGCCATTCCCGGCGACCCGAGAGACACCACCACGCCGCGGGCGATGGCGCAGACGTTGCGTCAGCTTACGCTGGGTCATGCGCTGGGCGAAACCCAGCGGGCGCAGTTGGTGACGTGGCTCAAAGGCAATACGACCGGCGCAGCCAGCATTCGGGCCGGCTTACCGACGTCGTGGACTGTGGGTGATAAGACCGGCAGCGGCGACTACGGCACCACCATG |
| CTX-10 | TATGTGCAGTACCAGTAAAGTTATGGCGGCCGCGGCGGTGCTTAAGCAGAGTGAAACGCAAAAGCAGCTGCTTAATCAGCCTGTCGAGATCAAGCCTGCCGATCTGGTTAACTACAATCCGATTGCCGAAAAACACGTCAACGGCACAATGACGCTGGCAGAACTGAGCGCGGCCGCGTTGCAGTACAGCGACAATACCGCCATGAACAAATTGATTGCCCAGCTCGGTGGCCCGGGAGGCGTGACGGCTTTTGCCCGCGCGATCGGCGATGAGACGTTTCGTCTGGATCGCACTGAACCTACGCTGAATACCGCCATTCCCGGCGACCCGAGAGACACCACCACGCCGCGGGCGATGGCGCAGACGTTGCGTCAGCTTACGCTGGGTCATGCGCTGGGCGAAACCCAGCGGGCGCAGTTGGTGACGTGGCTCAAAGGCAATACGACCGGCGCAGCCAGCATTCGGGCCGGCTTACCGACGTCGTGGACTGTGGGTGATAAGACCGGCAGCGGCGACTACGGCACCACCAATGATATCGCGGTA |
| CTX-14 | ATGTGCAGTACCAGTAAAGTTATGGCGGCCGCGGCGGTGCTTAAGCAGAGTGAAACGCAAAAGCAGCTGCTTAATCAGCCTGTCGAGATCAAGCCTGCCGATCTGGTTAACTACAATCCGATTGCCGAAAAACACGTCAACGGCACAATGACGCTGGCAGAACTGAGCGCGGCCGCGTTGCAGTACAGCGACAATACCGCCATGAACAAATTGATTGCCCAGCTCGGTGGCCCGGGAGGCGTGACGGCTTTTGCCCGCGCGATCGGCGATGAGACGTTTCGTCTGGATCGCACTGAACCTACGCTGAATACCGCCATTCCCGGCGACCCGAGAGACACCACCACGCCGCGGGCGATGGCGCAGACGTTGCGTCAGCTTACGCTGGGTCATGCGCTGGGCGAAACCCAGCGGGCGCAGTTGGTGACGTGGCTCAAAGGCAATACGACCGGCGCAGCCAGCATTCGGGCCGGCTTACCGACGTCGTGGACTGTGGGTGATAAGACCGGCAGCGGCGGCTACGGCACCACCAATGATAATCGCGGTA |
| CTX-15 | GCCCTTTTTTATGTGCAGTACCAGTAAAGTTATGGCGGCCGCGGCGGTGCTTAAGCAGAGTGAAACGCAAAAGCAGCTGCTTAATCAGCCTGTCGAGATCAAGCCTGCCGATCTGGTTAACTACAATCCGATTGCCGAAAAACACGTCAACGGCACAATGACGCTGGCAGAACTGAGCGCGGCCGCGTTGCAGTACAGCGACAATACCGCCATGAACAAATTGATTGCCCAGCTCGGTGGCCCGGGAGGCGTGACGGCTTTTGCCCGCGCGATCGGCGATGAGACGTTTCGTCTGGATCGCACTGAACCTACGCTGAATACCGCCATTCCCGGCGACCCGAGAGACACCACCACGCCGCGGGCGATGGCGCAGACGTTGCGTCAGCTTACGCTGGGTCATGCGCTGGGCGAAACCCAGCGGGCGCAGTTGGTGACGTGGCTCAAAGGCAATACGACCGGCGCAGCCAGCATTCGGGCCGGCTTACCGACGTCGTGGACTGTGGGTGATAAGACCGGCAGCGGCGACTACGGCACCACCAATGATATCGCGGT |
| CTX-26 | TTTTATGTGCAAGGCACAGTAAAGTTATGGCGGCCGCGGCGGTGCTTAAGCAGAGTGAAACGCAAAAGCAGCTGCTTAATCAGCCTGTCGAGATCAAGCCTGCCGATCTGGTTAACTACAATCCGATTGCCGAAAAACACGTCAACGGCACAATGACGCTGGCAGAACTGAGCGCGGCCGCGTTGCAGTACAGCGACAATACCGCCATGAACAAATTGATTGCCCAGCTCGGTGGCCCGGGAGGCGTGACGGCTTTTGCCCGCGCGATCGGCGATGAGACGTTTCGTCTGGATCGCACTGAACCTACGCTGAATACCGCCATTCCCGGCGACCCGAGAGACACCACCACGCCGCGGGCGATGGCGCAGACGTTGCGTCAGCTTACGCTGGGTCATGCGCTGGGCGAAACCCAGCGGGCGCAGTTGGTGACGTGGCTCAAAGGCAATACGACCGGCGCAGCCAGCATTCGGGCCGGCTTACCGACGTCGTGGACTGTGGGTGATAAGACCGGCAGCGGCGACTACGGCACCACCAATGTAAATCGCGGTA |
| CTX-27 | ATGTGCAGGTCCAGTAAAGTTATGGCGGCCGCGGCGGTGCTTAAGCAGAGTGAAACGCAAAAGCAGCTGCTTAATCAGCCTGTCGAGATCAAGCCTGCCGATCTGGTTAACTACAATCCGATTGCCGAAAAACACGTCAACGGCACAATGACGCTGGCAGAACTGAGCGCGGCCGCGTTGCAGTACAGCGACAATACCGCCATGAACAAATTGATTGCCCAGCTCGGTGGCCCGGGAGGCGTGACGGCTTTTGCCCGCGCGATCGGCGATGAGACGTTTCGTCTGGATCGCACTGAACCTACGCTGAATACCGCCATTCCCGGCGACCCGAGAGACACCACCACGCCGCGGGCGATGGCGCAGACGTTGCGTCAGCTTACGCTGGGTCATGCGCTGGGCGAAACCCAGCGGGCGCAGTTGGTGACGTGGCTCAAAGGCAATACGACCGGCGCAGCCAGCATTCGGGCCGGCTTACCGACGTCGTGGACTGTGGGTGATAAGACCGGCAGCGGCGACTACGGCACCACCAATGTAATCGCGGTAAAA |
| CTX-29 | ATGTGCAGTACCAGTAAAGTTATGGCGGCCGCGGCGGTGCTTAAGCAGAGTGAAACGCAAAAGCAGCTGCTTAATCAGCCTGTCGAGATCAAGCCTGCCGATCTGGTTAACTACAATCCGATTGCCGAAAAACACGTCAACGGCACAATGACGCTGGCAGAACTGAGCGCGGCCGCGTTGCAGTACAGCGACAATACCGCCATGAACAAATTGATTGCCCAGCTCGGTGGCCCGGGAGGCGTGACGGCTTTTGCCCGCGCGATCGGCGATGAGACGTTTCGTCTGGATCGCACTGAACCTACGCTGAATACCGCCATTCCCGGCGACCCGAGAGACACCACCACGCCGCGGGCGATGGCGCAGACGTTGCGTCAGCTTACGCTGGGTCATGCGCTGGGCGAAACCCAGCGGGCGCAGTTGGTGACGTGGCTCAAAGGCAATACGACCGGCGCAGCCAGCATTCGGGCCGGCTTACCGACGTCGTGGACTGTGGGTGATAAGACCGGCAGCGGCGACTACGGCACCACCAATGATATCGCGGTAAAGT |
| CTX-33 | ATGTGCAGTACCAGTAAAGTTATGGCGGCCGCGGCGGTGCTTAAGCAGAGTGAAACGCAAAAGCAGCTGCTTAATCAGCCTGTCGAGATCAAGCCTGCCGATCTGGTTAACTACAATCCGATTGCCGAAAAACACGTCAACGGCACAATGACGCTGGCAGAACTGAGCGCGGCCGCGTTGCAGTACAGCGACAATACCGCCATGAACAAATTGATTGCCCAGCTCGGTGGCCCGGGAGGCGTGACGGCTTTTGCCCGCGCGATCGGCGATGAGACGTTTCGTCTGGATCGCACTGAACCTACGCTGAATACCGCCATTCCCGGCGACCCGAGAGACACCACCACGCCGCGGGCGATGGCGCAGACGTTGCGTCAGCTTACGCTGGGTCATGCGCTGGGCGAAACCCAGCGGGCGCAGTTGGTGACGTGGCTCAAAGGCAATACGACCGGCGCAGCCAGCATTCGGGCCGGCTTACCGACGTCGTGGACTGTGGGTGATAAGACCGGCAGCGGCGACTACGGCACCACCAATGTAATCGCGGTA |
| CTX-42 | TTATGTGCAGGGTCCAGTAAAGTTATGGCGGCCGCGGCGGTGCTTAAGCAGAGTGAAACGCAAAAGCAGCTGCTTAATCAGCCTGTCGAGATCAAGCCTGCCGATCTGGTTAACTACAATCCGATTGCCGAAAAACACGTCAACGGCACAATGACGCTGGCAGAACTGAGCGCGGCCGCGTTGCAGTACAGCGACAATACCGCCATGAACAAATTGATTGCCCAGCTCGGTGGCCCGGGAGGCGTGACGGCTTTTGCCCGCGCGATCGGCGATGAGACGTTTCGTCTGGATCGCACTGAACCTACGCTGAATACCGCCATTCCCGGCGACCCGAGAGACACCACCACGCCGCGGGCGATGGCGCAGACGTTGCGTCAGCTTACGCTGGGTCATGCGCTGGGCGAAACCCAGCGGGCGCAGTTGGTGACGTGGCTCAAAGGCAATACGACCGGCGCAGCCAGCATTCGGGCCGGCTTACCGACGTCGTGGACTGTGGGTGATAAGACCGGCAGCGGCGACTACGGCACCACCAATGTAAATCGCGGTAA |
| CTX-45 | ATATGTGCAGTTCCAGTAAAGTTATGGCGGCCGCGGCGGTGCTTAAGCAGAGTGAAACGCAAAAGCAGCTGCTTAATCAGCCTGTCGAGATCAAGCCTGCCGATCTGGTTAACTACAATCCGATTGCCGAAAAACACGTCAACGGCACAATGACGCTGGCAGAACTGAGCGCGGCCGCGTTGCAGTACAGCGACAATACCGCCATGAACAAATTGATTGCCCAGCTCGGTGGCCCGGGAGGCGTGACGGCTTTTGCCCGCGCGATCGGCGATGAGACGTTTCGTCTGGATCGCACTGAACCTACGCTGAATACCGCCATTCCCGGCGACCCGAGAGACACCACCACGCCGCGGGCGATGGCGCAGACGTTGCGTCAGCTTACGCTGGGTCATGCGCTGGGCGAAACCCAGCGGGCGCAGTTGGTGACGTGGCTCAAAGGCAATACGACCGGCGCAGCCAGCATTCGGGCCGGCTTACCGACGTCGTGGACTGTGGGTGATAAGACCGGCAGCGGCGACTACGGCACCACCAATGTAATTCGCGGTAAAA |
| CTX-46 | ATGTGCAGGTCCAGTAAAGTTATGGCGGCCGCGGCGGTGCTTAAGCAGAGTGAAACGCAAAAGCAGCTGCTTAATCAGCCTGTCGAGATCAAGCCTGCCGATCTGGTTAACTACAATCCGATTGCCGAAAAACACGTCAACGGCACAATGACGCTGGCAGAACTGAGCGCGGCCGCGTTGCAGTACAGCGACAATACCGCCATGAACAAATTGATTGCCCAGCTCGGTGGCCCGGGAGGCGTGACGGCTTTTGCCCGCGCGATCGGCGATGAGACGTTTCGTCTGGATCGCACTGAACCTACGCTGAATACCGCCATTCCCGGCGACCCGAGAGACACCACCACGCCGCGGGCGATGGCGCAGACGTTGCGTCAGCTTACGCTGGGTCATGCGCTGGGCGAAACCCAGCGGGCGCAGTTGGTGACGTGGCTCAAAGGCAATACGACCGGCGCAGCCAGCATTCGGGCCGGCTTACCGACGTCGTGGACTGTGGGTGATAAGACCGGCAGCGGCGACTACGGCACCACCAATGTTAATCGCGGTA |
| CTX-47 | TTTTATGTGCAGTACCAGTAAAGTTATGGCGGCCGCGGCGGTGCTTAAGCAGAGTGAAACGCAAAAGCAGCTGCTTAATCAGCCTGTCGAGATCAAGCCTGCCGATCTGGTTAACTACAATCCGATTGCCGAAAAACACGTCAACGGCACAATGACGCTGGCAGAACTGAGCGCGGCCGCGTTGCAGTACAGCGACAATACCGCCATGAACAAATTGATTGCCCAGCTCGGTGGCCCGGGAGGCGTGACGGCTTTTGCCCGCGCGATCGGCGATGAGACGTTTCGTCTGGATCGCACTGAACCTACGCTGAATACCGCCATTCCCGGCGACCCGAGAGACACCACCACGCCGCGGGCGATGGCGCAGACGTTGCGTCAGCTTACGCTGGGTCATGCGCTGGGCGAAACCCAGCGGGCGCAGTTGGTGACGTGGCTCAAAGGCAATACGACCGGCGCAGCCAGCATTCGGGCCGGCTTACCGACGTCGTGGACTGTGGGTGATAAGACCGGCAGCGGCGACTACGGCACCACCAATGATATCGCGGT |
| TEM-5 | GGGTTTTTTCGGGTTTCGCCTTATTCCCTTTTTTGCGGCATTTTGCCTTCCTGTTTTTGCTCACCCAGAAACGCTGGTGAAAGTAAAAGATGCTGAAGATCAGTTGGGTGCACGAGTGGGTTACATCGAACTGGATCTCAACAGCGGTAAGATCCTTGAGAGTTTTCGCCCCGAAGAACGTTTTCCAATGATGAGCACTTTTAAAGTTCTGCTATGTGGTGCGGTATTATCCCGTGTTGACGCCGGGCAAGAGCAACTCGGTCGCCGCATACACTATTCTCAGAATGACTTGGTTGAGTACTCACCAGTCACAGAAAAGCATCTTACGGATGGCATGACAGTAAGAGAATTATGCAGTGCTGCCATAACCATGAGTGATAACACTGCTGCCAACTTACTTCTGACAACGATCGGAGGACCGAAGGAGCTAACCGCTTTTTTGCACAACATGGGGGATCATGTAACTCGCCTTGATCGTTGGGAACCGGAGCTGAATGAAGCCATACCAAACGACGAGCGTGACACCACGATGCCTGCAGCAATGGCAACAACGTTGCGCAAACTATTAACTGGCGAACTACTTACTCTAGCTTCCCGGCAACAATTAATAGACTGGATGGAGGCGGATAAAGTTGCAGGACCACTTCTGCGCTCGGCCCTTCCGGCTGGCTGGTTTATTGCTGAATTTGGGGGGACCCCGGGAGGGCGGCCCCG |
| TEM-6 | AATTTTTTTCGTGTCCGCCTTATTCCCTTTTTTGCGGCATTTTGCCTTCCTGTTTTTGCTCACCCAGAAACGCTGGTGAAAGTAAAAGATGCTGAAGATCAGTTGGGTGCACGAGTGGGTTACATCGAACTGGATCTCAACAGCGGTAAGATCCTTGAGAGTTTTCGCCCCGAAGAACGTTTTCCAATGATGAGCACTTTTAAAGTTCTGCTATGTGGTGCGGTATTATCCCGTGTTGACGCCGGGCAAGAGCAACTCGGTCGCCGCATACACTATTCTCAGAATGACTTGGTTGAGTACTCACCAGTCACAGAAAAGCATCTTACGGATGGCATGACAGTAAGAGAATTATGCAGTGCTGCCATAACCATGAGTGATAACACTGCTGCCAACTTACTTCTGACAACGATCGGAGGACCGAAGGAGCTAACCGCTTTTTTGCACAACATGGGGGATCATGTAACTCGCCTTGATCGTTGGGAACCGGAGCTGAATGAAGCCATACCAAACGACGAGCGTGACACCACGATGCCTGCAGCAATGGCAACAACGTTGCGCAAACTATTAACTGGCGAACTACTTACTCTAGCTTCCCGGCAACAATTAATAGACTGGATGGAGGCGGATAAAGTTGCAGGACCACTTCTGCGCTCGGCCCTTCCGGCTGGCTGGTTTATTGCTGATAAACTGGGGAGCCGGAAGGGCCGAGCGCAGAAGTGCTGGTGCTGCTTCTCTGTGCGACTCCCTCGGCTCTT |
| TEM-7 | CAATTTTTCGTGTTCCGCCTTATTCCCTTTTTTGCGGCATTTTGCCTTCCTGTTTTTGCTCACCCAGAAACGCTGGTGAAAGTAAAAGATGCTGAAGATCAGTTGGGTGCACGAGTGGGTTACATCGAACTGGATCTCAACAGCGGTAAGATCCTTGAGAGTTTTCGCCCCGAAGAACGTTTTCCAATGATGAGCACTTTTAAAGTTCTGCTATGTGGTGCGGTATTATCCCGTGTTGACGCCGGGCAAGAGCAACTCGGTCGCCGCATACACTATTCTCAGAATGACTTGGTTGAGTACTCACCAGTCACAGAAAAGCATCTTACGGATGGCATGACAGTAAGAGAATTATGCAGTGCTGCCATAACCATGAGTGATAACACTGCTGCCAACTTACTTCTGACAACGATCGGAGGACCGAAGGAGCTAACCGCTTTTTTGCACAACATGGGGGATCATGTAACTCGCCTTGATCGTTGGGAACCGGAGCTGAATGAAGCCATACCAAACGACGAGCGTGACACCACGATGCCTGCAGCAATGGCAACAACGTTGCGCAAACTATTAACTGGCGAACTACTTACTCTAGCTTCCCGGCAACAATTAATAGACTGGATGGAGGCGGATAAAGTTGCAGGACCACTTCTGCGCTCGGCCCTTCCGGCTGGCTGGTTTATTGCTGATAACCGGGGAAGCCGGAAGGCCGAGCGAGGG |
| TEM-8 | ATTTTTCGTGTTCCGCCTTATTCCCTTTTTTGCGGCATTTTGCCTTCCTGTTTTTGCTCACCCAGAAACGCTGGTGAAAGTAAAAGATGCTGAAGATCAGTTGGGTGCACGAGTGGGTTACATCGAACTGGATCTCAACAGCGGTAAGATCCTTGAGAGTTTTCGCCCCGAAGAACGTTTTCCAATGATGAGCACTTTTAAAGTTCTGCTATGTGGTGCGGTATTATCCCGTGTTGACGCCGGGCAAGAGCAACTCGGTCGCCGCATACACTATTCTCAGAATGACTTGGTTGAGTACTCACCAGTCACAGAAAAGCATCTTACGGATGGCATGACAGTAAGAGAATTATGCAGTGCTGCCATAACCATGAGTGATAACACTGCTGCCAACTTACTTCTGACAACGATCGGAGGACCGAAGGAGCTAACCGCTTTTTTGCACAACATGGGGGATCATGTAACTCGCCTTGATCGTTGGGAACCGGAGCTGAATGAAGCCATACCAAACGACGAGCGTGACACCACGATGCCTGCAGCAATGGCAACAACGTTGCGCAAACTATTAACTGGCGAACTACTTACTCTAGCTTCCCGGCAACAATTAATAGACTGGATGGAGGCGGATAAAGTTGCAGGACCACTTCTGCGCTCGGCCCTTCCGGCTGGCTGGTTTATTGCTGATAATCTGGGAAGCCGGAAG  GCCGAGCGCA |
| TEM-11 | TAAACACAAATTTTTCGTGTTCCGCCTTATTCCCTTTTTTGCGGCATTTTGCCTTCCTGTTTTTGCTCACCCAGAAACGCTGGTGAAAGTAAAAGATGCTGAAGATCAGTTGGGTGCACGAGTGGGTTACATCGAACTGGATCTCAACAGCGGTAAGATCCTTGAGAGTTTTCGCCCCGAAGAACGTTTTCCAATGATGAGCACTTTTAAAGTTCTGCTATGTGGTGCGGTATTATCCCGTGTTGACGCCGGGCAAGAGCAACTCGGTCGCCGCATACACTATTCTCAGAATGACTTGGTTGAGTACTCACCAGTCACAGAAAAGCATCTTACGGATGGCATGACAGTAAGAGAATTATGCAGTGCTGCCATAACCATGAGTGATAACACTGCTGCCAACTTACTTCTGACAACGATCGGAGGACCGAAGGAGCTAACCGCTTTTTTGCACAACATGGGGGATCATGTAACTCGCCTTGATCGTTGGGAACCGGAGCTGAATGAAGCCATACCAAACGACGAGCGTGACACCACGATGCCTGCAGCAATGGCAACAACGTTGCGCAAACTATTAACTGGCGAACTACTTACTCTAGCTTCCCGGCAACAATTAATAGACTGGATGGAGGCGGATAAAGTTGCAGGACCACTTCTGCGCTCGGCCCTTCCGGCTGGCTGGTTTATTGCTGATAAACTTGGGGAGCCCGGA |
| TEM-13 | TCCCAATTTTTCGTGTTCCGCCTTATTCCCTTTTTTGCGGCATTTTGCCTTCCTGTTTTTGCTCACCCAGAAACGCTGGTGAAAGTAAAAGATGCTGAAGATCAGTTGGGTGCACGAGTGGGTTACATCGAACTGGATCTCAACAGCGGTAAGATCCTTGAGAGTTTTCGCCCCGAAGAACGTTTTCCAATGATGAGCACTTTTAAAGTTCTGCTATGTGGTGCGGTATTATCCCGTGTTGACGCCGGGCAAGAGCAACTCGGTCGCCGCATACACTATTCTCAGAATGACTTGGTTGAGTACTCACCAGTCACAGAAAAGCATCTTACGGATGGCATGACAGTAAGAGAATTATGCAGTGCTGCCATAACCATGAGTGATAACACTGCTGCCAACTTACTTCTGACAACGATCGGAGGACCGAAGGAGCTAACCGCTTTTTTGCACAACATGGGGGATCATGTAACTCGCCTTGATCGTTGGGAACCGGAGCTGAATGAAGCCATACCAAACGACGAGCGTGACACCACGATGCCTGCAGCAATGGCAACAACGTTGCGCAAACTATTAACTGGCGAACTACTTACTCTAGCTTCCCGGCAACAATTAATAGACTGGATGGAGGCGGATAAAGTTGCAGGACCACTTCTGCGCTCGGCCCTTCCGGCTGGCTGGTTTATTGCTGATAACCTGGGGGACCCCGGA |
| TEM-14 | CCCTTTTTTGGGTGTTCCCCTTATTCCCTTTTTTGCGGCATTTTGCCTTCCTGTTTTTGCTCACCCAGAAACGCTGGTGAAAGTAAAAGATGCTGAAGATCAGTTGGGTGCACGAGTGGGTTACATCGAACTGGATCTCAACAGCGGTAAGATCCTTGAGAGTTTTCGCCCCGAAGAACGTTTTCCAATGATGAGCACTTTTAAAGTTCTGCTATGTGGTGCGGTATTATCCCGTGTTGACGCCGGGCAAGAGCAACTCGGTCGCCGCATACACTATTCTCAGAATGACTTGGTTGAGTACTCACCAGTCACAGAAAAGCATCTTACGGATGGCATGACAGTAAGAGAATTATGCAGTGCTGCCATAACCATGAGTGATAACACTGCTGCCAACTTACTTCTGACAACGATCGGAGGACCGAAGGAGCTAACCGCTTTTTTGCACAACATGGGGGATCATGTAACTCGCCTTGATCGTTGGGAACCGGAGCTGAATGAAGCCATACCAAACGACGAGCGTGACACCACGATGCCTGCAGCAATGGCAACAACGTTGCGCAAACTATTAACTGGCGAACTACTTACTCTAGCTTCCCGGCAACAATTAATAGACTGGATGGAGGCGGATAAAGTTGCAGGACCACTTCTGCGCTCGGCCCTTCCGGCTGGCTGGTTTATTGCTGAAATTGGGGGGACCCGGGAGGCGGCGCGCGG |
| TEM-15 | AATTTTTCGTGTTCCGCCTTATTCCCTTTTTTGCGGCATTTTGCCTTCCTGTTTTTGCTCACCCAGAAACGCTGGTGAAAGTAAAAGATGCTGAAGATCAGTTGGGTGCACGAGTGGGTTACATCGAACTGGATCTCAACAGCGGTAAGATCCTTGAGAGTTTTCGCCCCGAAGAACGTTTTCCAATGATGAGCACTTTTAAAGTTCTGCTATGTGGTGCGGTATTATCCCGTGTTGACGCCGGGCAAGAGCAACTCGGTCGCCGCATACACTATTCTCAGAATGACTTGGTTGAGTACTCACCAGTCACAGAAAAGCATCTTACGGATGGCATGACAGTAAGAGAATTATGCAGTGCTGCCATAACCATGAGTGATAACACTGCTGCCAACTTACTTCTGACAACGATCGGAGGACCGAAGGAGCTAACCGCTTTTTTGCACAACATGGGGGATCATGTAACTCGCCTTGATCGTTGGGAACCGGAGCTGAATGAAGCCATACCAAACGACGAGCGTGACACCACGATGCCTGCAGCAATGGCAACAACGTTGCGCAAACTATTAACTGGCGAACTACTTACTCTAGCTTCCCGGCAACAATTAATAGACTGGATGGAGGCGGATAAAGTTGCAGGACCACTTCTGCGCTCGGCCCTTCCGGCTGGCTGGTTTATTGCTGATAAACTTGGGGAAGCCCGGA |
| TEM-18 | AATTTTTCGTGTTCCGCCTTATTCCCTTTTTTGCGGCATTTTGCCTTCCTGTTTTTGCTCACCCAGAAACGCTGGTGAAAGTAAAAGATGCTGAAGATCAGTTGGGTGCACGAGTGGGTTACATCGAACTGGATCTCAACAGCGGTAAGATCCTTGAGAGTTTTCGCCCCGAAGAACGTTTTCCAATGATGAGCACTTTTAAAGTTCTGCTATGTGGTGCGGTATTATCCCGTGTTGACGCCGGGCAAGAGCAACTCGGTCGCCGCATACACTATTCTCAGAATGACTTGGTTGAGTACTCACCAGTCACAGAAAAGCATCTTACGGATGGCATGACAGTAAGAGAATTATGCAGTGCTGCCATAACCATGAGTGATAACACTGCTGCCAACTTACTTCTGACAACGATCGGAGGACCGAAGGAGCTAACCGCTTTTTTGCACAACATGGGGGATCATGTAACTCGCCTTGATCGTTGGGAACCGGAGCTGAATGAAGCCATACCAAACGACGAGCGTGACACCACGATGCCTGCAGCAATGGCAACAACGTTGCGCAAACTATTAACTGGCGAACTACTTACTCTAGCTTCCCGGCAACAATTAATAGACTGGATGGAGGCGGATAAAGTTGCAGGACCACTTCTGCGCTCGGCCCTTCCGGCTGGCTGGTTTATTGCTGATAAACTTGGGGAAGCCCGGA |
| TEM-21 | ATAAAGTTGCAGGACCACTTCTGCGCCCGGCCCTTCCGGGTTTCCAAGTTATCAGCAATAAACCAGCCAGCCGGAAGGGCCGAGCGCAGAAGTGGTCCTGCAACTTTATCCGCCTCCATCCAGTCTATTAATTGTTGCCGGGAAGCTAGAGTAAGTAGTTCGCCAGTTAATAGTTTGCGCAACGTTGTTGCCATTGCTGCAGGCATCGTGGTGTCACGCTCGTCGTTTGGTATGGCTTCATTCAGCTCCGGTTCCCAACGATCAAGGCGAGTTACATGATCCCCCATGTTGTGCAAAAAAGCGGTTAGCTCCTTCGGTCCTCCGATCGTTGTCAGAAGTAAGTTGGCAGCAGTGTTATCACTCATGGTTATGGCAGCACTGCATAATTCTCTTACTGTCATGCCATCCGTAAGATGCTTTTCTGTGACTGGTGAGTACTCAACCAAGTCATTCTGAGAATAGTGTATGCGGCGACCGAGTTGCTCTTGCCCGGCGTCAACACGGGATAATACCGCACCACATAGCAGAACTTTAAAAGTGCTCATCATTGGAAAACGTTCTTCGGGGCGAAAACTCTCAAGGATCTTACCGCTGTTGAGATCCAGTTCGATGTAACCCACTCGTGCACCCAACTGATCTTCAGCATCTTTTACTTTCACCAGCGTTTCTGGGTGAGCAAAAACAGGAAGGCAAAATGCCGCAAAAAAGGGAATAAGGGGGACACGAAAAGGGG |
| TEM-23 | CTTCCGGCTCTCCCAAGTTATCAGCAATAAACCAGCCAGCCGGAAGGGCCGAGCGCAGAAGTGGTCCTGCAACTTTATCCGCCTCCATCCAGTCTATTAATTGTTGCCGGGAAGCTAGAGTAAGTAGTTCGCCAGTTAATAGTTTGCGCAACGTTGTTGCCATTGCTGCAGGCATCGTGGTGTCACGCTCGTCGTTTGGTATGGCTTCATTCAGCTCCGGTTCCCAACGATCAAGGCGAGTTACATGATCCCCCATGTTGTGCAAAAAAGCGGTTAGCTCCTTCGGTCCTCCGATCGTTGTCAGAAGTAAGTTGGCAGCAGTGTTATCACTCATGGTTATGGCAGCACTGCATAATTCTCTTACTGTCATGCCATCCGTAAGATGCTTTTCTGTGACTGGTGAGTACTCAACCAAGTCATTCTGAGAATAGTGTATGCGGCGACCGAGTTGCTCTTGCCCGGCGTCAACACGGGATAATACCGCACCACATAGCAGAACTTTAAAAGTGCTCATCATTGGAAAACGTTCTTCGGGGCGAAAACTCTCAAGGATCTTACCGCTGTTGAGATCCAGTTCGATGTAACCCACTCGTGCACCCAACTGATCTTCAGCATCTTTTACTTTCACCAGCGTTTCTGGGTGAGCAAAAACAGGAAGGCAAAATGCCGCAAAAAAGGGAATAAGGGCGACACGAAAA |
| TEM-29 | GGCTTTTTTTTTTGGGTTTTCCCCTTATTCCCTTTTTTGCGGCATTTTGCCTTCCTGTTTTTGCTCACCCAGAAACGCTGGTGAAAGTAAAAGATGCTGAAGATCAGTTGGGTGCACGAGTGGGTTACATCGAACTGGATCTCAACAGCGGTAAGATCCTTGAGAGTTTTCGCCCCGAAGAACGTTTTCCAATGATGAGCACTTTTAAAGTTCTGCTATGTGGTGCGGTATTATCCCGTGTTGACGCCGGGCAAGAGCAACTCGGTCGCCGCATACACTATTCTCAGAATGACTTGGTTGAGTACTCACCAGTCACAGAAAAGCATCTTACGGATGGCATGACAGTAAGA  GAATTATGCAGTGCTGCCATAACCATGAGTGATAACACTGCTGCCAACTTACTTCTGACAACGATCGGAGGACCGAAGGAGCTAACCGCTTTTTTGCACAACATGGGGGATCATGTAACTCGCCTTGATCGTTGGGAACCGGAGCTGAATGAAGCCATACCAAACGACGAGCGTGACACCACGATGCCTGCAGCAATGGCAACAACGTTGCGCAAACTATTAACTGGCGAACTACTTACTCTAGCTTCCCGGCAACAATTAATAGACTGGATGGAGGCGGATAAAGTTGCAGGACCACTTCTGCGCTCGGCCCTTCCGGCTGGCTGGTTTATTGCTGATAAACTGGGGAGCCGGAAGG |
| TEM-30 | CCCCTTTTCGTGTTCCCCTTATTCCCTTTTTTGCGGCATTTTGCCTTCCTGTTTTTGCTCACCCAGAAACGCTGGTGAAAGTAAAAGATGCTGAAGATCAGTTGGGTGCACGAGTGGGTTACATCGAACTGGATCTCAACAGCGGTAAGATCCTTGAGAGTTTTCGCCCCGAAGAACGTTTTCCAATGATGAGCACTTTTAAAGTTCTGCTATGTGGTGCGGTATTATCCCGTGTTGACGCCGGGCAAGAGCAACTCGGTCGCCGCATACACTATTCTCAGAATGACTTGGTTGAGTACTCACCAGTCACAGAAAAGCATCTTACGGATGGCATGACAGTAAGAGAATTATGCAGTGCTGCCATAACCATGAGTGATAACACTGCTGCCAACTTACTTCTGACAACGATCGGAGGACCGAAGGAGCTAACCGCTTTTTTGCACAACATGGGGGATCATGTAACTCGCCTTGATCGTTGGGAACCGGAGCTGAATGAAGCCATACCAAACGACGAGCGTGACACCACGATGCCTGCAGCAATGGCAACAACGTTGCGCAAACTATTAACTGGCGAACTACTTACTCTAGCTTCCCGGCAACAATTAATAGACTGGATGGAGGCGGATAAAGTTGCAGGACCACTTCTGCGCTCGGCCCTTCCGGCTGGCTGGTTTATTGCTGATAAATCTTGGAGCCGGA |
| TEM-31 | GGCTTTTCGTGTTCCCCTTATTCCCTTTTTTGCGGCATTTTGCCTTCCTGTTTTTGCTCACCCAGAAACGCTGGTGAAAGTAAAAGATGCTGAAGATCAGTTGGGTGCACGAGTGGGTTACATCGAACTGGATCTCAACAGCGGTAAGATCCTTGAGAGTTTTCGCCCCGAAGAACGTTTTCCAATGATGAGCACTTTTAAAGTTCTGCTATGTGGTGCGGTATTATCCCGTGTTGACGCCGGGCAAGAGCAACTCGGTCGCCGCATACACTATTCTCAGAATGACTTGGTTGAGTACTCACCAGTCACAGAAAAGCATCTTACGGATGGCATGACAGTAAGAGAATTATGCAGTGCTGCCATAACCATGAGTGATAACACTGCTGCCAACTTACTTCTGACAACGATCGGAGGACCGAAGGAGCTAACCGCTTTTTTGCACAACATGGGGGATCATGTAACTCGCCTTGATCGTTGGGAACCGGAGCTGAATGAAGCCATACCAAACGACGAGCGTGACACCACGATGCCTGCAGCAATGGCAACAACGTTGCGCAAACTATTAACTGGCGAACTACTTACTCTAGCTTCCCGGCAACAATTAATAGACTGGATGGAGGCGGATAAAGTTGCAGGACCACTTCTGCGCTCGGCCCTTCCGGCTGGCTGGTTTATTGCTGATAAACTGGGAAGCCGGAAG |
| TEM-13 | CTTCTGCGCCCGGCCCTTCCGGGTTTCCCAGATATCAGCAATAAACCAGCCAGCCGGAAGGGCCGAGCGCAGAAGTGGTCCTGCAACTTTATCCGCCTCCATCCAGTCTATTAATTGTTGCCGGGAAGCTAGAGTAAGTAGTTCGCCAGTTAATAGTTTGCGCAACGTTGTTGCCATTGCTGCAGGCATCGTGGTGTCACGCTCGTCGTTTGGTATGGCTTCATTCAGCTCCGGTTCCCAACGATCAAGGCGAGTTACATGATCCCCCATGTTGTGCAAAAAAGCGGTTAGCTCCTTCGGTCCTCCGATCGTTGTCAGAAGTAAGTTGGCAGCAGTGTTATCACTCATGGTTATGGCAGCACTGCATAATTCTCTTACTGTCATGCCATCCGTAAGATGCTTTTCTGTGACTGGTGAGTACTCAACCAAGTCATTCTGAGAATAGTGTATGCGGCGACCGAGTTGCTCTTGCCCGGCGTCAACACGGGATAATACCGCACCACATAGCAGAACTTTAAAAGTGCTCATCATTGGAAAACGTTCTTCGGGGCGAAAACTCTCAAGGATCTTACCGCTGTTGAGATCCAGTTCGATGTAACCCACTCGTGCACCCAACTGATCTTCAGCATCTTTTACTTTCACCAGCGTTTCTGGGTGAGCAAAAACAGGAAGGCAAAATGCCGCAAAAAAGGGAATAAGGGGAAAAC |
| TEM-36 | CTTTTCGTGTCGCCCTTATTCCCTTTTTTGCGGCATTTTGCCTTCCTGTTTTTGCTCACCCAGAAACGCTGGTGAAAGTAAAAGATGCTGAAGATCAGTTGGGTGCACGAGTGGGTTACATCGAACTGGATCTCAACAGCGGTAAGATCCTTGAGAGTTTTCGCCCCGAAGAACGTTTTCCAATGATGAGCACTTTTAAAGTTCTGCTATGTGGTGCGGTATTATCCCGTGTTGACGCCGGGCAAGAGCAACTCGGTCGCCGCATACACTATTCTCAGAATGACTTGGTTGAGTACTCACCAGTCACAGAAAAGCATCTTACGGATGGCATGACAGTAAGAGAATTATGCAGTGCTGCCATAACCATGAGTGATAACACTGCTGCCAACTTACTTCTGACAACGATCGGAGGACCGAAGGAGCTAACCGCTTTTTTGCACAACATGGGGGATCATGTAACTCGCCTTGATCGTTGGGAACCGGAGCTGAATGAAGCCATACCAAACGACGAGCGTGACACCACGATGCCTGCAGCAATGGCAACAACGTTGCGCAAACTATTAACTGGCGAACTACTTACTCTAGCTTCCCGGCAACAATTAATAGACTGGATGGAGGCGGATAAAGTTGCAGGACCACTTCTGCGCTCGGCCCTTCCGGCTGGCTGGTTTATTGCTGATAAATCTGGAGCCGGAAGGGCCGAGCGCAGAA |
| TEM-41 | GCACCAATTAAAGGACTGGATGGAGGCGGATAAAGTTGCAGGACCACTTCTGCGCTCGGCCCTTCCGGCTCCAGATTTATCAGCAATAAACCAGCCAGCCGGAAGGGCCGAGCGCAGAAGTGGTCCTGCAACTTTATCCGCCTCCATCCAGTCTATTAATTGTTGCCGGGAAGCTAGAGTAAGTAGTTCGCCAGTTAATAGTTTGCGCAACGTTGTTGCCATTGCTGCAGGCATCGTGGTGTCACGCTCGTCGTTTGGTATGGCTTCATTCAGCTCCGGTTCCCAACGATCAAGGCGAGTTACATGATCCCCCATGTTGTGCAAAAAAGCGGTTAGCTCCTTCGGTCCTCCGATCGTTGTCAGAAGTAAGTTGGCAGCAGTGTTATCACTCATGGTTATGGCAGCACTGCATAATTCTCTTACTGTCATGCCATCCGTAAGATGCTTTTCTGTGACTGGTGAGTACTCAACCAAGTCATTCTGAGAATAGTGTATGCGGCGACCGAGTTGCTCTTGCCCGGCGTCAACACGGGATAATACCGCACCACATAGCAGAACTTTAAAAGTGCTCATCATTGGAAAACGTTCTTCGGGGCGAAAACTCTCAAGGATCTTACCGCTGTTGAGATCCAGTTCGATGTAACCCACTCGTGCACCCAACTGATCTTCAGCATCTTTTACTTTCACCAGCGTTTCTGGGTGAGCAAAAACAGGAAGGCAAAATGCCGCAAAAAAGGGAATAAGGGCGACACGAAAAC |
| TEM-46 | CAGCGAGAACTACTTTTCGTGTCGCCCTTATTCCCTTTTTTGCGGCATTTTGCCTTCCTGTTTTTGCTCACCCAGAAACGCTGGTGAAAGTAAAAGATGCTGAAGATCAGTTGGGTGCACGAGTGGGTTACATCGAACTGGATCTCAACAGCGGTAAGATCCTTGAGAGTTTTCGCCCCGAAGAACGTTTTCCAATGATGAGCACTTTTAAAGTTCTGCTATGTGGTGCGGTATTATCCCGTGTTGACGCCGGGCAAGAGCAACTCGGTCGCCGCATACACTATTCTCAGAATGACTTGGTTGAGTACTCACCAGTCACAGAAAAGCATCTTACGGATGGCATGACAGTAAGAGAATTATGCAGTGCTGCCATAACCATGAGTGATAACACTGCTGCCAACTTACTTCTGACAACGATCGGAGGACCGAAGGAGCTAACCGCTTTTTTGCACAACATGGGGGATCATGTAACTCGCCTTGATCGTTGGGAACCGGAGCTGAATGAAGCCATACCAAACGACGAGCGTGACACCACGATGCCTGCAGCAATGGCAACAACGTTGCGCAAACTATTAACTGGCGAACTACTTACTCTAGCTTCCCGGCAACAATTAATAGACTGGATGGAGGCGGATAAAGTTGCAGGACCACTTCTGCGCTCGGCCCTTCCGGCTGGCTGGTTTATTGCTGATAAATCTGG  AGCCGGAAG |
| TEM-48 | GTTTTTCCGTGTTTCGCCTTATTCCCTTTTTTGCGGCATTTTGCCTTCCTGTTTTTGCTCACCCAGAAACGCTGGTGAAAGTAAAAGATGCTGAAGATCAGTTGGGTGCACGAGTGGGTTACATCGAACTGGATCTCAACAGCGGTAAGATCCTTGAGAGTTTTCGCCCCGAAGAACGTTTTCCAATGATGAGCACTTTTAAAGTTCTGCTATGTGGTGCGGTATTATCCCGTGTTGACGCCGGGCAAGAGCAACTCGGTCGCCGCATACACTATTCTCAGAATGACTTGGTTGAGTACTCACCAGTCACAGAAAAGCATCTTACGGATGGCATGACAGTAAGAGAATTATGCAGTGCTGCCATAACCATGAGTGATAACACTGCTGCCAACTTACTTCTGACAACGATCGGAGGACCGAAGGAGCTAACCGCTTTTTTGCACAACATGGGGGATCATGTAACTCGCCTTGATCGTTGGGAACCGGAGCTGAATGAAGCCATACCAAACGACGAGCGTGACACCACGATGCCTGCAGCAATGGCAACAACGTTGCGCAAACTATTAACTGGCGAACTACTTACTCTAGCTTCCCGGCAACAATTAATAGACTGGATGGAGGCGGATAAAGTTGCAGGACCACTTCTGCGCTCGGCCCTTCCGGCTGGCTGGTTTATTGCTGATAACTTGGAAACCCGGAGGGGCCGAGCGCAGAAGTGGTCCTGCAGCTTTATCC |
| TEM-49 | TATAATACAAACTTTTTCGTGTCGCCCTTATTCCCTTTTTTGCGGCATTTTGCCTTCCTGTTTTTGCTCACCCAGAAACGCTGGTGAAAGTAAAAGATGCTGAAGATCAGTTGGGTGCACGAGTGGGTTACATCGAACTGGATCTCAACAGCGGTAAGATCCTTGAGAGTTTTCGCCCCGAAGAACGTTTTCCAATGATGAGCACTTTTAAAGTTCTGCTATGTGGTGCGGTATTATCCCGTGTTGACGCCGGGCAAGAGCAACTCGGTCGCCGCATACACTATTCTCAGAATGACTTGGTTGAGTACTCACCAGTCACAGAAAAGCATCTTACGGATGGCATGACAGTAAGAGAATTATGCAGTGCTGCCATAACCATGAGTGATAACACTGCTGCCAACTTACTTCTGACAACGATCGGAGGACCGAAGGAGCTAACCGCTTTTTTGCACAACATGGGGGATCATGTAACTCGCCTTGATCGTTGGGAACCGGAGCTGAATGAAGCCATACCAAACGACGAGCGTGACACCACGATGCCTGCAGCAATGGCAACAACGTTGCGCAAACTATTAACTGGCGAACTACTTACTCTAGCTTCCCGGCAACAATTAATAGACTGGATGGAGGCGGATAAAGTTGCAGGACCACTTCTGCGCTCGGCCCTTCCGGCTGGCTGGTTTATTGCTGATAAATCTGG  GAGCCGGAAGGG |
| TEM-50 | ACCACTTCTGCGCTCGGCCCTTCCGGCTTCCAAGTTTATCAGCAATAAACCAGCCAGCCGGAAGGGCCGAGCGCAGAAGTGGTCCTGCAACTTTATCCGCCTCCATCCAGTCTATTAATTGTTGCCGGGAAGCTAGAGTAAGTAGTTCGCCAGTTAATAGTTTGCGCAACGTTGTTGCCATTGCTGCAGGCATCGTGGTGTCACGCTCGTCGTTTGGTATGGCTTCATTCAGCTCCGGTTCCCAACGATCAAGGCGAGTTACATGATCCCCCATGTTGTGCAAAAAAGCGGTTAGCTCCTTCGGTCCTCCGATCGTTGTCAGAAGTAAGTTGGCAGCAGTGTTATCACTCATGGTTATGGCAGCACTGCATAATTCTCTTACTGTCATGCCATCCGTAAGATGCTTTTCTGTGACTGGTGAGTACTCAACCAAGTCATTCTGAGAATAGTGTATGCGGCGACCGAGTTGCTCTTGCCCGGCGTCAACACGGGATAATACCGCACCACATAGCAGAACTTTAAAAGTGCTCATCATTGGAAAACGTTCTTCGGGGCGAAAACTCTCAAGGATCTTACCGCTGTTGAGATCCAGTTCGATGTAACCCACTCGTGCACCCAACTGATCTTCAGCATCTTTTACTTTCACCAGCGTTTCTGGGTGAGCAAAAACAGGAAGGCAAAATGCCGCAAAAAAGGGAATAAGGGCGACACGAAAA |
| SHV-9 | TTTTGGGTATATTTTTTTTTTGTCGCTTCTTTACTCGCCTTTATCGGCCCTCACTCAAGGATGTATTGTGGTTATGCGTTATATTCGCCTGTGTATTATCTCCCTGTTAGCCACCCTGCCGCTGGCGGTTCACGCCAGCCCGCAGCCGCTTGAGCAAATTAAACAAAGCGAAAGCCAGCTGTCGGGCCGCGTAGGCATGATAGAAATGGATCTGGCCAGCGGCCGCACGCTGACCGCCTGGCGCGCCGATGAACGCTTTCCCATGATGAGCACCTTTAAAGTAGTGCTCTGCGGCGCAGTGCTGGCGCGGGTGGATGCCGGTGACGAACAGCTGGAGCGAAAGATCCACTATCGCCAGCAGGATCTGGTGGACTACTCGCCGGTCAGCGAAAAACACCTTGCCGACGGCATGACGGTCGGCGAACTCTGTGCCGCCGCCATTACCATGAGCGATAACAGCGCCGCCAATCTGCTGCTGGCCACCGTCGGCGGCCCCGCAGGATTGACTGCCTTTTTGCGCCAGATCGGCGACAACGTCACCCGCCTTGACCGCTGGGAAACGGAACTGAATGAGGCGCTTCCCGGCGACGCCCGCGACACCACTACCCCGGCCAGCATGGCCGCGACCCTGCGCAAGCTGCTGACCAGCCAGCGTCTGAGCGCCCGTTCGCAACGGCAGCTGCTGCAGTGGATGGTGGACGATCGGGTCGCCGGACCGTTGATCCGCTCCGTGCTGCCGGCGGGCTGGTTTATCGCCGATAAGACCGGAGCTGGCGAACGGGGTGCGCGCGGGATTGTCGCCCTGCTTGGCCCGAATAACAAAGCAGAGCGCATCGTGGTGATTTATCTGCGGGATACGCCGGCGAGCATGGCCGAGCGAAATCAGCAAATCGCCGGGATCGGCGCGGCGCTGATCGAGATGGGGAAAACGCTAAAAA |
| SHV-42 | TTTTGGGGATATTTTTTTTTGTCGCTTCTTTACTCGCCTTTATCGGCCCTCACTCAAGGATGTATTGTGGTTATGCGTTATATTCGCCTGTGTATTATCTCCCTGTTAGCCACCCTGCCGCTGGCGGTTCACGCCAGCCCGCAGCCGCTTGAGCAAATTAAACAAAGCGAAAGCCAGCTGTCGGGCCGCGTAGGCATGATAGAAATGGATCTGGCCAGCGGCCGCACGCTGACCGCCTGGCGCGCCGATGAACGCTTTCCCATGATGAGCACCTTTAAAGTAGTGCTCTGCGGCGCAGTGCTGGCGCGGGTGGATGCCGGTGACGAACAGCTGGAGCGAAAGATCCACTATCGCCAGCAGGATCTGGTGGACTACTCGCCGGTCAGCGAAAAACACCTTGCCGACGGCATGACGGTCGGCGAACTCTGTGCCGCCGCCATTACCATGAGCGATAACAGCGCCGCCAATCTGCTGCTGGCCACCGTCGGCGGCCCCGCAGGATTGACTGCCTTTTTGCGCCAGATCGGCGACAACGTCACCCGCCTTGACCGCTGGGAAACGGAACTGAATGAGGCGCTTCCCGGCGACGCCCGCGACACCACTACCCCGGCCAGCATGGCCGCGACCCTGCGCAAGCTGCTGACCAGCCAGCGTCTGAGCGCCCGTTCGCAACGGCAGCTGCTGCAGTGGATGGTGGACGATCGGGTCGCCGGACCGTTGATCCGCTCCGTGCTGCCGGCGGGCTGGTTTATCGCCGATAAGACCGGAGCTGGCGAACGGGGTGCGCGCGGGATTGTCGCCCTGCTTGGCCCGAATAACAAAGCAGAGCGCATCGTGGTGATTTATCTGCGGGATACGCCGGCGAGCATGGCCGAGCGAAATCAGCAAATCGCCGGGATCGGCGCGGCGCTGATCGACCTGGTGAAACACCCTA |
| SHV-49 | TTTTGGGTTATTTTTTTATTGTCGCGAAATGGAATTTCATCAAAATTATAGAAATAGCAGTTATCGTACCAGGGCTTACCTTTAACCAGGGTGGTGAGGTATTCATACATGCCTTTGGGCGTGCTACCGGCGGTAATTGCCAGGTTAACACGACGCGTCTTCGACATATAACCAAGCAGATGGTGTGCCGCGACACGGCTCATTTCCTGGTAATCTTCGGTAATGATTAATTTCATCTTAATGCCTTATTTTATTTAAATAATTTCTGTACAAAGGCTGCATAGGCTGGTCGCCAGACATCCATTTCATGGTTCAGACCCGGATATTCCTGGTAATCAAAGTTAATTTTTTTCTGCTCAAGCTCAGTTTTCAGCCCGGCGATATCCTTGCCGGTTACGACATCTTTATCCCCCACAACCACAGTAAAATTACGTAGTTGCTGGTTGATAGCTGCCGGATCGTTCAGTCGGGCCGCGACACCTTCATCCGGTACGGTTGTCGTGGTAACACCACTGAATGTGGCCAGCCAGCCAAAGCTTTCCAGATGATTCATTCCGGAAACCAGCGCCTGGTACCCGCCTTGTGAAAGCCCTGCCAGCGCGCGGCCATCGGCATCTTTACGGACATTAAAACGCTTGCTAATCAGCGGGATAATATCGTTCATCAGTTCGCGATCTGCCGCTTTAGCATTCAGCGGATAAAAGACTTTACGTCTTTCCTGAGGCACGAAATCTTCGGGAATAATGCCCTTCGCATCGGTTTCTGTATCCGGGATCACCACCAGCATCGGTTTAATTTTCCCTTCAGCAAGCAGGTTATCCATGATTTGCGGGATACGCCCCTGATCGATAGCGGAACGTCCGGTATCACCAAAGCCGTGATAGAAATAGAGCATGGCCAACGGCTAAAAA |
